# Molecular Mechanisms Associated with Plant Tolerance upon Abiotic Stress

**DOI:** 10.3390/plants13243532

**Published:** 2024-12-18

**Authors:** Emilia L. Apostolova

**Affiliations:** Institute of Biophysics and Biomedical Engineering, Bulgarian Academy of Sciences, Acad. G. Bonchev Str., Bl. 21, 1113 Sofia, Bulgaria; emya@bio21.bas.bg or emilia.apostolova@gmail.com

## Abstract

The important processes of plants are influenced by adverse environmental factors, which can have a negative impact on their growth and development. The last decade has seen an increase in the impact of abiotic stress on plants due to climate changes. The impact of abiotic stress on plants and their defense mechanisms is presented in the Special Issue “Molecular Mechanisms Associated with Plant Tolerance upon Abiotic Stress”. The studies enhance our understanding of how abiotic factors affect plants and their defense mechanisms.

## 1. Introduction

The abiotic stress factors of the environment, such as drought, salinity, extreme temperatures, and others, are significantly increased by climate change [1,2]. In nature, plants are exposed to multiple stress conditions, and adaptation to these stressors involves integrative responses to individual stressors and the formation of a new type of response [3]. The effects of abiotic stress factors vary depending on the type of stress, its duration, and also the plant species [4,5,6,7]. Furthermore, it has been shown that there are variations in how stressors are tolerated between different varieties of a specific species [8,9]. These stresses greatly affect photosynthesis in plants. The reduction in yield is directly related to the decrease in the photosynthetic capacity of plants due to environmental stress [10]. For adaptation to multiple stresses, it is very important to have a transcription factor that can bind to the promoters of downstream genes [11]. Zinc finger-homeodomain (ZF-HD) proteins are specific transcription factors in plants that play an important role in their growth, development, and stress responses [12]. A recent genome-wide analysis of ZF-HD genes in *Phyllostachys edulis* has identified the key candidate genes related to abiotic stress in this plant [13].

## 2. Salt Stress

Salinity causes osmotic stress and ion toxicity in plants, which leads to oxidative stress. The overproduction of reactive oxygen species (ROS) under salt stress damage nucleic acids, proteins, lipids, and other macromolecules [14,15]. The activity of ROS triggers the membrane lipid peroxidation, chlorophyll degradation, and changes in the membrane integrity and its fluidity [16,17], which leads to changes in the structural arrangement of chloroplasts and modifications of the pigment-protein complexes of the thylakoid membranes [8,18]. Recent research revealed the important role of the rearrangement of mitochondrial metabolism for the adjustment of plant cells under salt stress. It has been shown that the regulation of glutamate metabolism through epigenetic mechanisms involves coordinated changes in the activities of 2-oxoglutarate-dehydrogenase, glutamate dehydrogenase, and glutamate decarboxylase [19].

The study of the adaptive mechanisms of *Poa pratensis* L. in saline environments revealed an increased expression of genes associated with chlorophyll biosynthesis, components of the complex of photosystem II (PSII), the Calvin cycle, and those involved in the biosynthesis of ROS-scavenging enzymes [20]. The authors also revealed a key role of the accumulation of proline and betaine as well as the upregulation of genes involved in Na^+^/K^+^ transport (such as HKT1;5, HAK5, and SKOR) in improving the salt tolerance of *Poa pratensis* L. [20].

The influence of salt stress on different plant species was used to assess the reasons/mechanisms for the salt tolerance of plants. Previous studies revealed that salt-induced changes in the photosynthetic apparatus decrease in the open PSII reaction centers, influence the electron flow from Q_A_^−^ to plastoquinone, and diminish the effective quantum yield of PSII, which were more strongly influenced in plant-sensitive species [6,7]. Moreover, the increase in energy losses after high salt treatment of more tolerant plant species is a result of enhancement mainly in regulated energy losses, while in the sensitive species, non-regulated energy losses occur [6,7]. The study of the impact of salinity on photosynthetic pigments, ions, osmolytes, oxidative stress markers, and antioxidant metabolites, as well as antioxidant enzyme activities in the *Imperata cylindrica*, *Phragmites australis*, and *Saccharum ravennae*, showed different sensitivities of these plant species [21]. The authors concluded that the tolerance mechanism to salinity is based on the inhibition of the Na^+^ and Cl^−^ transport from roots to the aerial part of the plant and the accumulation of proline and soluble sugars in the photosynthetic tissues. It has been suggested that the activation of K^+^ transport in leaves under salt stress contributes to an increase in salt tolerance [21].

Nitric oxide (NO) plays a significant role in the plant response to abiotic stress [22]. Nitric oxide acts as an antioxidant against ROS under stress and also can act as a signal molecule [23]. Previous research showed that NO alleviated the salt-induced changes in growth, development, and functions of the plants [24,25]. Recently, it has been shown that NO prevents changes in the fluidity of thylakoid membranes, energy transfer between the pigment protein complexes of the photosynthetic apparatus, and modification of the Mn clusters of the oxygen-evolving complex in the donor side of the PSII complex [17]. The impact of NO on the acceptor side of PSII was also registered, influencing Q_A_^−^ reoxidation. In plants treated with SNP under salt stress, Q_A_^−^ interaction with plastoquinone is more dominant than the recombination of electrons in Q_A_Q_B_^−^ with the oxidized S_2_ (S_3_) state of the oxygen-evolving complex. The impact of the NO on the donor and acceptor side of the PSII under salt stress corresponded with better PSII efficiency and alleviation of the photosynthetic performance under salt stress [17].

## 3. Drought Stress

Global food production is negatively impacted by drought stress, a natural climatic phenomenon [26]. Plant development and crop production depend heavily on photosynthetic performance, which is significantly impacted by this stress [27]. The drought-induced changes in cell metabolism correspond with the overproduction of ROS, which disrupts proteins, lipids, and cell membranes [28]. Previous studies showed the changes in the composition, organization, and functions of the complexes of the photosynthetic apparatus depend on the drought tolerance of plant species [29,30]. A recent study with drought-susceptible and drought-tolerant wheat varieties revealed higher osmolyte accumulation and left water content in the tolerant variety in comparison to the variety that is sensitive under drought stress [31]. Moreover, it has been shown to increase antioxidant enzyme activity, which decreases membrane damage under drought. Proteomic analysis showed that drought induced the differential expression of proteins involved in biological processes in both wheat varieties (drought-tolerant and drought-susceptible) [31]. It has been shown that tolerant variety reprogramming protein synthesis in order to obtain the proteins with regulatory and/or protection roles against the effects of low water content. Moloi et al. [31] suggested that the observed downregulation of the proteins involved in photosynthesis in tolerant varieties might be a protective mechanism against oxidative stress in plants under drought. Increased levels of stress markers and non-enzymatic compounds as well as the activities of antioxidative and xenobiotic-detoxifying enzymes in triticale (*Triticum* spp.) under drought were shown [32].

The use of biostimulants in agriculture, as a key factor in the stimulation of plant growth and the enhancement of crop production under physiological and stress conditions, has increased [33,34]. In recent years, there has been increasing interest in the effects of melatonin on plants to prevent adverse environmental effects. Melatonin plays an important role in the integration of various environmental signals and the formation of the plant stress response [35]. It regulates the expression of the photosynthetic genes, preserves the chlorophyll molecules and photosynthetic functions, and interacts with NO and ROS to regulate the plant redox network [36,37]. Foliar spraying of oregano (*Origanum vulgare* L.) plants with 100 µM melatonin under moderate drought stress ameliorates PSII photochemistry [38]. It is suggested that under these conditions, melatonin triggered a non-photochemical mechanism to decrease ^1^O_2_ production, which resulted in an increase in the open PSII centers and an increase in the electron transport rate. The effect of melatonin under drought depends on the plant species and light intensity, suggesting research with different plant species before the extensive use of melatonin in agriculture [38].

## 4. Cold Stress

Cold stress has harmful effects on plant growth, development, and their yield. Plants develop different mechanisms to copy this stress and induce a cold tolerance from the cellular level to the whole plant level [39]. Researchers demonstrate that NAC genes play an important role in controlling the cold tolerance in plants [40,41]. The study of Yang et al. [42] identified a total of 110 NAC genes in *Dendrobium officinale*, which were classified in 15 subfamilies. In addition, cold-responsive DoNACs were validated.

The study of the impacts of low temperature and low light on the wild type and mutant (tangerine) of *Solanum lycopersicum* L. demonstrates that the oxidative stress in both types of plants is higher after combined treatment with low temperature and low light in comparison to the low light only [43]. The author also revealed the protective role of the phenolic components, including anthocyanins, against cold-induced ROS.
